# Clinging ability is related to particular aspects of foot morphology in salamanders

**DOI:** 10.1002/ece3.7888

**Published:** 2021-07-17

**Authors:** Erica K. Baken, Mary Kate O’Donnell

**Affiliations:** ^1^ Department of Science Chatham University Pittsburgh PA USA; ^2^ Department of Ecology and Evolutionary Biology Brown University Providence RI USA

**Keywords:** arboreality, clinging, performance, salamander, shape

## Abstract

The interaction between morphology, performance, and ecology has long been studied in order to explain variation in the natural world. Within arboreal salamanders, diversification in foot morphology and microhabitat use are thought to be linked by the impact of foot size and shape on clinging and climbing performance, resulting in an ability to access new habitats. We examine whether various foot shape metrics correlate with stationary cling performance and microhabitat to explicitly quantify this performance gradient across 14 species of salamander, including both arboreal and nonarboreal species. Clinging performance did not correlate with foot shape, as quantified by landmark‐based geometric morphometrics, nor with microhabitat use. Mass‐corrected foot centroid size and foot contact area, on the other hand, correlated positively with clinging performance on a smooth substrate. Interestingly, these foot variables correlated negatively with clinging performance on rough substrates, suggesting the use of multiple clinging mechanisms dependent upon the texture of the surface. These findings demonstrate that centroid size and foot contact area are more functionally relevant for clinging in salamanders than foot shape, suggesting that foot shape need not converge in order to achieve convergent performance. More broadly, our results provide an example of how the quantification of the performance gradient can provide the appropriate lens through which to understand the macroevolution of morphology and ecology.

## Graphical abstract

Arboreal salamanders have confounded herpetologists for years. We test the relationship between form and function of foot shape with respect to arboreality, showing no dependence of clinging performance on foot shape or microhabitat use. Rather, mass‐corrected foot centroid size and foot contact area do confer differences in cling performance.

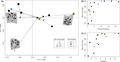

## 1 INTRODUCTION

Identifying the ecological pressures that influence morphological evolution is of primary interest in evolutionary biology. Associations between ecological and morphological traits indicate how species interact with their environment and are interpreted as evidence of natural selection. For instance, the repeated evolution of specialized body proportions in arboreal *Anolis* lizards suggests that the biomechanical constraints of arboreal life drive morphological diversity across these clades (Losos, 1990). However, to draw conclusions about the evolution of morphology and ecology, one must assume that morphological variation confers variation in relevant performance measures. Arnold's (1983) ecomorphological paradigm emphasizes the importance of performance in mediating evolutionary mechanisms; without a connection to relevant performance, morphology has limited impact on fitness and thus cannot be under strong selection. Only by elucidating the relationships between morphology, ecology, *and* performance can we understand how ecology and evolution interact to shape a clade of interest.

In systems that demonstrate a close relationship between morphology and ecology, the assumed role of performance is often examined to hone the details of how morphology confers relevant performance variation. The above example of *Anolis* lizards spurred subsequent experiments that revealed how each ecomorph confers a different performance specialization corresponding to their specific microhabitat (Harmon et al., 2005; Losos et al., 1998). This approach provided a full story of how these lizards interact with their environment and how microhabitat selection drove morphological adaptations through locomotion performance, display behaviors, and perch preferences. On the other hand, some systems display a surprising disconnect between morphology, performance, and ecology. For example, Douglas and Matthews (1992) tested the relationship between diet and morphology across 17 fish species while considering phylogeny and revealed that morphology was related to phylogenetic history but, surprisingly, not diet. In these scenarios, it is unclear exactly where the ecomorphological paradigm breaks down: Does morphology *not* confer variation in performance, does performance *not* confer variation in fitness, or both? Or does morphology impact fitness via performance metrics that were not captured by the experiment? For Douglas and Matthews (1992), a narrowing of the phylogenetic scope to a single family revealed the expected morphological and ecological correlations, and follow‐up studies demonstrated that body shape and prey capture performance were, as expected, closely tied in certain species (Rincón et al., 2007). In this case, the disconnect observed by Douglas and Matthews (1992) had been caused by the scope of the initial investigation, not aspects of the ecomorphological paradigm itself. In this study, we explore the long‐held assumption that foot morphology predictably corresponds to microhabitat use in salamanders, which recent work has called into question (Baken & Adams, 2019). We explicitly tested the relationships between morphology, performance, and microhabitat use, with the goal of identifying the step along which this paradigm, taken at a macroevolutionary scale, has broken down.

Salamanders (order: Caudata; 742 sp.; AmphibiaWeb, 2020) display a wide range of body sizes (25 mm–1 m; Parra‐Olea et al., 2016; Petranka, 1998), life histories (e.g., oviparous, direct developing, kleptogenesis; Petranka, 1998), morphological specializations (e.g., ballistic tongues: Lombard & Wake, 1977; prehensile tails: Garman, 1886; ), and microhabitat types (e.g., arboreal, terrestrial, troglodytic, fossorial, aquatic; Petranka, 1998). Along these ecological axes, several intriguing relationships between salamander morphology and ecology have been discovered. For example, Bonett and Blair (2017) observed rapid body elongation in clades that had recolonized the aquatic microhabitat. Adams and Nistri (2010) demonstrated ontogenetic convergence of webbed toes in a cave‐dwelling European clade, *Hydromantes*. In the same vein, many herpetologists have long assumed a functional role of foot shape in determining landscape movement, performance, and microhabitat use.

Many species of salamander occupy arboreal habitats year‐round, including members of the genera *Aneides*, *Bolitoglossa*, *Chiropterotriton*, *Dendrotriton*, *Ixalotrition*, *Nototriton*, *Pseudoeurycea*, and *Thorius* (McEntire, 2016; Spickler et al., 2006; Wake, 1987). Even primarily terrestrial and semi‐aquatic species have been observed clinging to and climbing up tree trunks, plant stems, cave walls, talus slopes, and vertical rock faces (Bradley & Eason, 2018; Camp et al., 2013; Crawford & Peterman, 2013; Gorman & Camp, 2006; Huheey & Brandon, 1973; Lunghi et al., 2017; McEntire, 2016; Spickler et al., 2006; Waldron & Humphries, 2005). Up to 45% of nonfully aquatic plethodontid salamanders have been documented climbing in their natural environments (McEntire, 2016). Reasons for engaging in climbing up and clinging on plants and rocks include finding food (Jaeger, 1978; Legros, 2013) or escaping larger predatory species (Crawford & Peterman, 2013). Elevated, sheltered habitats or caves also form a vital refuge from unfavorable temperature or humidity conditions for these desiccation‐prone species (Forsman & Swingl, 2007; Gorman & Camp, 2006; Lunghi et al., 2017; Spickler et al., 2006; Wake 2014) and may be used in nesting (Lunghi et al., 2014, 2015; Myer, 1958; Spickler et al., 2006; Waldron & Humphries, 2005). Thus, it is clear that access to these climbing‐accessible environments provides numerous benefits across salamander species.

Historically, claims have been made that certain foot shapes have evolved in salamanders as adaptations to arboreal life (e.g., Alberch, 1981; Taylor, 1944; Wake & Brame, 1969; Wake & Lynch, 1976). Jaekel and Wake (2007) later concluded that webbed toes in Bolitoglossans were a consequence of paedomorphosis, conferring a selective advantage in only a few lineages in which it is observed. In contrast, their analysis of the pattern of ontogenetic foot growth in the cave‐dwelling species *Chiropterotriton magnipes* suggested selection for increased foot surface area, which they inferred would increase clinging and climbing performance (Jaekel & Wake, 2007). Despite long‐standing interest in this topic, a clear relationship between foot shape, the actual performance it confers, and the macroevolutionary consequences of microhabitat use on that relationship have not been fully investigated.

Recent studies on clinging performance demonstrate that a salamander's ability to cling to an inclined surface may be accomplished by two distinct mechanisms: species cling to smooth surfaces through friction and adhesion, or they cling to coarse surfaces by grabbing onto or interlocking digits into the grooves of the surface (O’Donnell & Deban, 2020a, 2020b; Wang et al., 2016). Specialization for each of these mechanisms would likely result in a loss of clinging performance via the opposite mechanisms; that is to say, friction and adhesion can be improved by increasing foot area, most easily achieved via increasing toe webbing, whereas the clinging mechanism could be enhanced by elongation and increased dexterity in slender digits. Thus, the evolution of foot shape across salamanders, were it to follow selective pressures of clinging performance, could manifest a variety of macroevolutionary patterns. For instance, we might predict to find distinct climbing foot shape ecomorphs depending upon the microhabitat substrate, one for species more dependent upon clinging to leaves displaying more webbed toes and another for species more dependent upon clinging to the trunk and branches of trees displaying distinctly unwebbed toes. Yet, in an extensive investigation into foot shape evolution, Baken and Adams (2019) found no discernable macroevolutionary pattern indicating specialization toward any arboreal ecomorphs. This suggests that either (a) foot shape does not influence clinging performance of either style, (b) clinging performance has not experienced strong selection in arboreal plethodontids, or both.

In this study, we directly test whether foot shape correlates with clinging performance across arboreal and nonarboreal species by quantifying foot shape, smooth and rough surface clinging performance, and microhabitat use for 14 salamander species (Table 1). Using a subset of the foot shape and microhabitat data collected by Baken and Adams (2019), additional metrics of foot shape (foot centroid size, foot contact area), and clinging performance measured across a variety of surface substrates (O’Donnell & Deban, 2020b), we reveal the precise nature of how shape, performance, and microhabitat use relate in this system, contradicting long‐held assumptions in the field. In so doing, we present a clear explanation as to why salamander foot shape and arboreality are not correlated across macroevolutionary time as expected: Foot shape does not directly confer performance variation relevant for life in trees, but rather foot centroid size and foot contact area do. This study reveals that shape, per se, need not conform to a single morphotype to have converged upon a single functional advantage. Our findings also help reframe our understanding of how salamanders interact with their environment, giving rise to several new avenues of exploration into the relationship between salamander body morphology, clinging mechanics, and the evolutionary mechanisms that drive various axes of salamander diversity. In so doing, we demonstrate the necessity of Arnold's ecomorphological paradigm when untangling complex relationships between ecology and evolution.

**TABLE 1:** Microhabitat classifications, body mass (grams), and clinging performance data

Species	*n*	Microhabitat	Body mass	Max cling angle (S)	Max cling angle (R)
*Ambystoma maculatum*	5	T	28.80 ± 3.05	99 ± 5	138 ± 4
*Aneides flavipunctatus*	5	T	3.30 ± 2.38	141 ± 6	175 ± 5
*Aneides lugubris*	4	A	11.16 ± 0.41	144 ± 7	180 ± 0
*Aneides vagrans*	7	A	2.10 ± 1.01	169 ± 7	180 ± 0
*Bolitoglossa franklini*	9	A	3.29 ± 0.75	174 ± 4	163 ± 6
*Desmognathus aeneus*	5	T	0.36 ± 0.04	180 ± 0	168 ± 8
*Desmognathus ocoee*	5	T	0.88 ± 0.17	178 ± 2	173 ± 7
*Desmognathus quadramaculatus*	6	W	7.60 ± 0.98	132 ± 14	134 ± 10
*Ensatina eschscholtzii*	5	T	6.38 ± 0.60	109 ± 8	171 ± 6
*Eurycea guttolineata*	2	T	0.86 ± 0.31	180 ± 0	160 ± 20
*Eurycea wilderae*	5	W	0.60 ± 0.06	180 ± 0	176 ± 4
*Plethodon elongatus*	6	T	2.31 ± 0.27	175 ± 5	143 ± 5
*Plethodon metcalfi*	5	T	3.42 ± 0.30	161 ± 2	151 ± 4
*Pseudotriton ruber*	4	T	11.66 ± 0.47	148 ± 10	136 ± 7

Note

Microhabitat use is classified as arboreal (A), terrestrial (T), or aquatic (W), indicating a species primary microhabitat preference. Body mass and maximum clinging angle (°) on smooth (S) and rough (R) surfaces are represented as species' means ± standard error of the mean across *n* individuals.

## 2 METHODS

### 2.1 Microhabitat use

To elucidate the relationship between foot morphology, clinging performance, and microhabitat use, we examined 14 species of salamanders (Table 1). We classified adult microhabitat use (as it pertains to occupied substrate; i.e., tree canopy versus pond) from published literature, accounts from field observations, species descriptions, and other natural history sources (AmphibiaWeb, 2020; IUCN, 2010; McEntire, 2016; Petranka, 1998). Classification procedures followed Baken and Adams (2019) with the exception of the fossorial species, *Ambystoma maculatum,* included in the terrestrial category.

### 2.2 Clinging performance

We used a subset of the cling performance data from O’Donnell and Deban (2020b) to sample salamander cling performance on smooth and rough (asperity size 2,000–4,000 μm) epoxy‐resin substrates. Salamanders were placed on the substrate at 0° (horizontal; oriented head up after rotation), allowed to acclimate for a period of 30 s, and then rotated at a rate of three degrees/second. The angle at which the salamander detached from the surface was recorded, up to an angle of 180° (see O’Donnell & Deban, 2020b for more details). The order of species and individuals was randomized for each trial. All trials were conducted at 84 ± 10% humidity at 16–18°C. Data collected included the body mass and cling performance from 73 individuals (mean = 5.21 individuals/species), and the maximum performance from each individual in five trials was used in generating species means (Table 1).

### 2.3 Foot shape

We tested three metrics of foot morphology against microhabitat use and clinging performance: landmark‐based geometric morphometric full shape, centroid size of that full shape, and foot contact area. For the first two metrics, we used a subset of the foot shape data from Baken and Adams (2019), where right hind‐foot shape was quantified using two‐dimensional landmark‐based geometric morphometrics (Adams et al., 2013; Bookstein, 1991). Using 11 fixed landmarks and 10 semilandmarks, we quantified foot shape variation, capturing toe length, toe spread, toepad width, and interdigital webbing (see Baken & Adams, 2019 for more details). Semilandmarks were allowed to slide between bracketing landmarks by minimizing bending energy. These data represent 136 specimens (mean = 9.7 individuals/species), aligned using the generalized Procrustes procedure to remove nonshape variation of position, rotation, and scale before taking species means. During this procedure, we also extracted species mean centroid size as another metric of foot shape as it represents the amount of surface area that could potentially be used to adhere to the substrate.

Species average foot contact area (FCA) and body mass were drawn from 98 individuals (mean = 7 individuals/species) representing a subset of the surface area and cling performance data in O’Donnell and Deban (2020a). The portion of the right hind‐foot in close adhesive contact with a smooth acrylic surface at 0° (horizontal) was illuminated using frustrated total internal reflection and quantified in ImageJ (as described in Betts et al., 1980). This measure quantifies the area of the foot that contributes to attachment in vivo. Comparison of contact area at 0° and maximum cling angle has previously shown that while small gains or losses of contact area are possible with increasing angle, salamanders are not increasing maximum cling performance by the addition of contact area during rotation (O’Donnell & Deban, 2020a). As a result, data were collected on FCA for all species at 0°. Area data for each individual were drawn from the single trial in which they achieved the highest cling angle and also had their feet in contact with the substrate (which was not always the case in maximal cling performances) (O’Donnell & Deban, 2020a). From this dataset, body mass was also collected for inclusion in our analyses.

### 2.4 Analysis

We first examined the relationship between clinging ability and foot shape for both smooth and rough surfaces using two‐block partial least squares (cling_angle ~ foot_shape + phy), implemented with the function “phylo.integration” in the R package (R Core Team, 2021), geomorph (“two.b.pls” does not allow for the incorporation of phylogenetic relatedness; Adams et al., 2021). Then, to test the relationship between foot shape, cling performance, and microhabitat type, we performed the same analyses on the data subsets defined by microhabitat type (e.g., cling_angle ~ shape within arboreal species). For the centroid size and FCA, we tested the mass‐corrected values against maximum cling angle for smooth and rough surfaces across microhabitat types while accounting for phylogenetic relatedness using an ANCOVA model (cling_angle ~ (centroid_size/mass) * microhabitat + phy and cling_angle ~ (FCA/mass) * microhabitat + phy, respectively), implemented with the geomorph function “procD.pgls.” Finally, to ensure that the above ANCOVA patterns related to mass‐corrected foot variables were not solely driven by variation in body size (i.e., mass), we tested an alternative model that included mass as a random variable (e.g., cling_angle ~ (centroid_size/mass) * microhabitat + mass + phy).

All analyses used the Caudata phylogeny from Bonett and Blair (2017) to account for variation due to relatedness, pruned to match the species in this study. We determined significance using residual randomization permutation procedures (Adams & Collyer, 2018) with 1,000 iterations.

## 3 RESULTS

**FIGURE 1:**
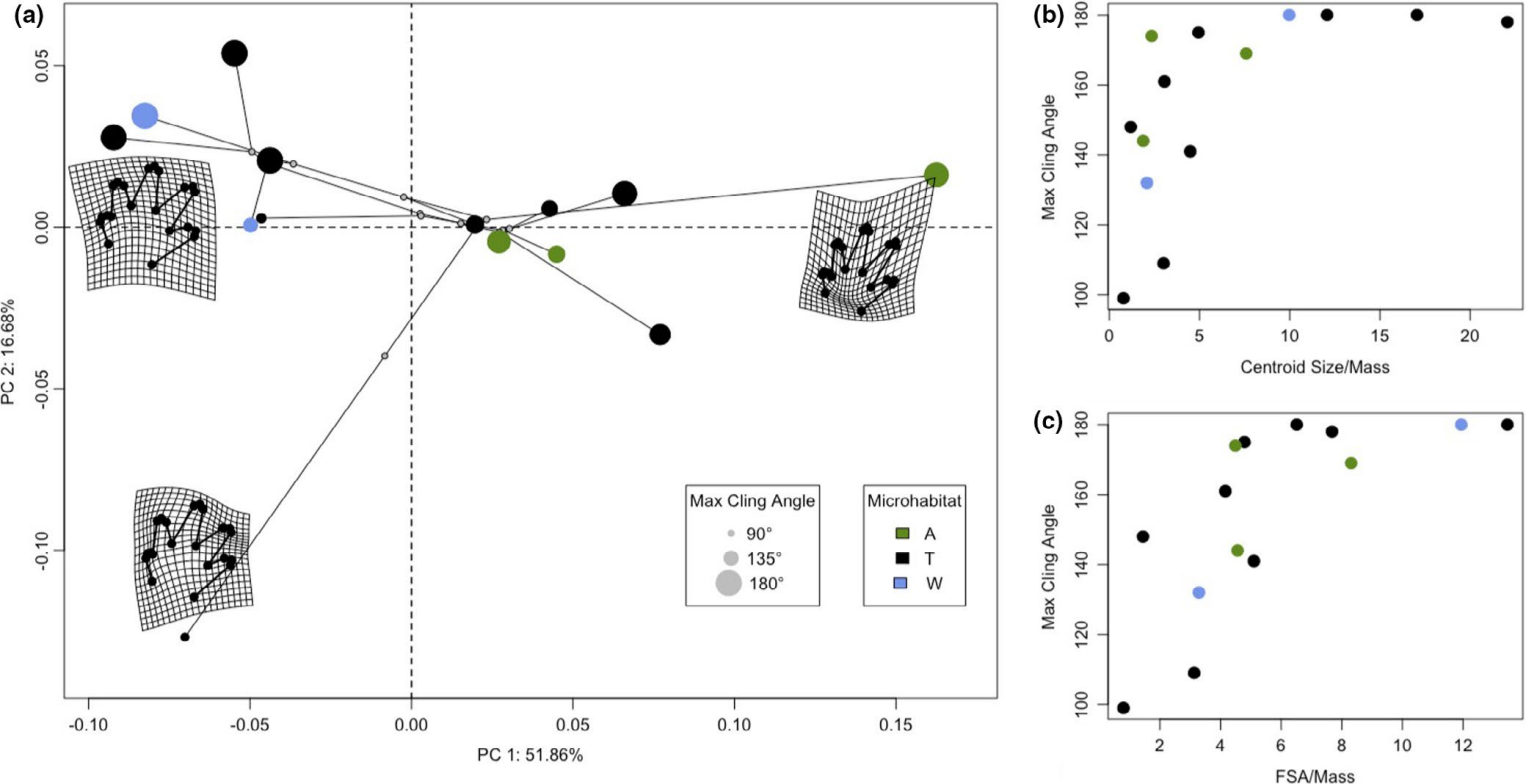
a. Phylomorphospace of foot shape characterized using geometric morphometrics. The size of the points corresponds to max cling angle. Warp grids represent the shape averages of select specimens (magnitude = 1). b. Max cling angle on a smooth surface across mean centroid size corrected for mass. c. Max cling angle on a smooth surface across foot contact area corrected for mass (FCA)

We did not observe any significant relationships between foot shape, microhabitat type, and clinging ability on either smooth or rough surfaces when using the landmark‐based geometric morphometric quantification of foot shape (*p* > 0.251; Table 2; Figure 1a). However, all ANCOVAs examining mass‐corrected centroid size and foot contact area (FCA) revealed significant, positive relationships between the foot variable and maximum cling angle on the smooth surface (Z > 2.2512; *p* < 0.007; Table 3). The rough surface results also produced significant results (Z > 1.9096; *p* < 0.025; Table 3), yet the estimated coefficients revealed a negative relationship between the mass‐corrected foot variable and maximum cling angle (CS/mass slope = −0.9388; FCA/mass slope = −1.0541). Although not shown in the main text, these patterns were also found in the analyses that did not include mass as a random variable (Table S1). None of the results showed a significant microhabitat term, indicating that clinging performance did not differ across microhabitat types (*p* > 0.266; Table 3).

**TABLE 2:** Two‐block partial least squares results regarding foot shape (landmark‐based geometric morphometrics), clinging performance across rough and smooth surfaces, and microhabitat

Substrate	Data Subset	r‐PLS	*Z*	*p* Value
Smooth	Full	0.6294	0.5579	0.285
	A	0.8813	0.2923	0.424
	T	0.6812	−0.1805	0.587
	W	1.0000	0.9900	0.501
Rough	Full	0.6294	0.5579	0.285
	A	0.9644	1.2208	0.251
	T	0.6950	0.0034	0.494
	W	1.0000	0.9900	0.501

Note

The first row for each substrate represents the analyses performed on the full dataset, and the subsequent rows are results from data subset by microhabitat type. No terms were significant for these analyses.

**TABLE 3:** ANCOVA results of mass‐corrected foot centroid size and foot contact area on clinging performance

Substrate	Term	DF	SS	MS	*Z*	*p* Value
Smooth	**CS/mass**	**1**	**72.4643**	**72.4643**	**2.2512**	0.**007**
	Microhabitat	2	18.0937	9.0469	0.5463	0.302
	Mass	1	29.7516	29.7516	1.5023	0.062
	(CS/mass)*Microhabitat	2	3.9032	1.9516	−0.5090	0.674
	Residuals	7	43.8372	6.2625		
	Total	13	168.0501			
	**FCA/mass**	**1**	**85.3187**	**85.3187**	**2.3241**	0.**006**
	Microhabitat	2	13.1452	6.5726	0.2028	0.422
	Mass	1	19.3496	19.3496	1.1970	0.109
	(FCA/mass)*Microhabitat	2	3.6903	1.8451	−0.5831	0.729
	Residuals	7	46.5463	6.6495		
	Total	13	168.0501			
Rough	**CS/mass**	**1**	**20.4076**	**20.4076**	**1.9096**	0.**025**
	Microhabitat	2	7.8709	3.9354	0.6872	0.266
	Mass	1	2.7505	2.7505	0.5466	0.307
	(CS/mass)*Microhabitat	**2**	**24.0031**	**12.0015**	**1.8570**	0.**029**
	Residuals	7	16.5265	2.3609		
	Total	13	71.5585			
	**FCA/mass**	**1**	**24.1249**	**24.1249**	**1.9303**	0.**021**
	Microhabitat	2	6.4910	3.2455	0.4764	0.305
	Mass	1	0.8055	0.8055	−0.1141	0.558
	(FCA/mass)*Microhabitat	**2**	**23.0532**	**11.5266**	**1.8277**	0.**033**
	Residuals	7	17.0840	2.4406		
	Total	13	71.5585			

Note

Significant terms, as determined by a *p* value below .05, are bolded. Both mass‐corrected centroid size (CS/mass) and mass‐corrected foot contact area (FCA/mass) were significantly related to maximum cling angle on smooth and rough surfaces. For the smooth surface, this relationship is positive (CS/mass slope = 0.6452; FCA/mass slope = 1.1938). On the rough surfaces, the model coefficients indicate a negative relationship between foot variable and clinging ability (CS/mass slope = −0.9388; FCA/mass slope = −1.0541). There are also significant interaction terms between the foot variable and microhabitat on rough surfaces, indicating that the negative correlation mentioned above varies in slope between microhabitat types. Consistently, the term for microhabitat use was not significant (*p* >  0 .266). *R* ^2^ and *F* values were omitted to conserve space, and the results of the ANCOVA excluding mass as a random variable can be found in the Supporting Information (Table S1).

## 4 DISCUSSION

The ecomorphological paradigm is a useful tool for explaining a wide variety of ecological and evolutionary patterns. It can be particularly illuminating when applied to systems with surprising disconnects between performance, ecology, and evolution (Koehl, 1996). In this study, we applied the ecomorphological paradigm to such a system: salamanders' apparent lack of foot shape specialization in arboreal species (Baken & Adams, 2019). We tested the two previously unexplored prongs of this morphology–performance–ecology relationship: whether foot shape confers clinging ability and whether clinging ability is stronger in arboreal species. Our data demonstrate that clinging ability is not correlated with microhabitat use (Table 3), and foot shape quantified by landmark‐based geometric morphometrics is not significantly related to clinging performance or microhabitat use on either smooth or rough surfaces (Table 2). We instead revealed that mass‐corrected centroid size and foot contact area (FCA) correlate significantly with clinging performance, however in different directions depending on the substrate to which they are clinging; maximum cling angle increases with higher mass‐corrected centroid size and FCA on smooth surfaces, yet the opposite relationship exists on rough surfaces (Figure 1b,c; Table 3). Overall, this approach revealed that foot shape affects clinging performance, but only insomuch as it determines centroid size and potential FCA. Put another way, a wide variety of different foot shapes may confer similar variation in the functionally relevant traits of centroid size and FCA. As such, this manuscript demonstrates the specific nature of how salamander morphology, performance, and microhabitat use relate in the context of the ecomorphological paradigm. Much of the remaining discussion explores potential future avenues of investigation that could build on these findings.

One of the more interesting results of this study was the contrasting functional performance of centroid size and FCA across surface types. That clinging ability increases with mass‐corrected centroid size and FCA on smooth surfaces, but decreases on rough surfaces corresponds with what has already been demonstrated about the mechanisms of clinging to these different surfaces. On smooth surfaces, friction and adhesion are the main mechanisms for clinging performance, which are accomplishable via greater available (centroid size) and used (FCA) foot surface area per unit mass. However, larger foot surface area would not necessarily allow for grabbing onto or interlocking digits into the grooves of the surface, as is the mechanism for clinging to rough surfaces (O’Donnell & Deban, 2020a, 2020b; Wang et al., 2016). This result suggests that both centroid size and FCA are conducive to friction and adhesion‐based clinging at the cost of dexterity. Thus, species that require the ability to navigate both smooth and rough surfaces may not be able to optimize clinging in both scenarios. One potential avenue of investigation would be to employ the analytical approach presented by Ghalambor et al. (2003) to quantify how a trait with multiple, potentially competing, functional roles responds to natural selection. Such previous studies have helped explain the evolution of multifaceted morphology–performance relationships (e.g., Moen, 2019), and employing such an approach in this system could lead to a better understanding of how foot shape confers performance across substrate type.

Of note is the relatively small number of species used in this study, including the fact that only three arboreal species were represented. This could result in a loss of power for detecting significant differences in clinging ability across microhabitat types. There are also several species of salamander that display a greater degree of webbing than those included in this study. However, previous investigations into the evolutionary dynamics of microhabitat use reveal that these three species represent at least two independent transitions toward arboreality (Baken & Adams, 2019), one occurring in the tropical tribe, *Bolitoglossini*, and another occurring in the North American genus, *Aneides*. Since the same study concluded that the number of independent transitions toward arboreality could be as low as five, our inclusion of two independent events lends confidence to our results. Further expanding these data to include more arboreal species, although it may prove logistically challenging, could reveal new or different patterns with respect to microhabitat. However, our results show that if such differences exist between arboreal and nonarboreal species, it is not a strong enough pattern as to be detectable at this level of taxon sampling.

An important path for future research is asking whether climbing ability rather than clinging ability is affected by foot shape variation. Climbing locomotion may present challenges distinct from those caused by clinging in the form of reduced contact area of attachment and the use of different body surfaces than those used in stationary clinging. The need for attachment and detachment cycling during climbing gaits requires the formation of strong but temporary attachment between the animal and the surface, but in some cases, climbing performance exceeds clinging performance. In the case of geckos, animals appear to outperform stationary clinging because they move faster than they slip (Stark et al., 2015). Regarding salamanders, foot shape and foot area may not represent the main drivers of climbing performance because of the role of the prehensile tails (present in some species; Petranka, 1998) or the differences in the duration for which attachment is needed. Alternately, foot shape (and the underlying musculoskeletal structures) may be critical to determining foot use during climbing, including strength and dexterity of gripping attachment or in fine‐scale adjustments in foot placement, loading, and foot peeling behaviors similar to those found in geckos. Further investigation in the kinematics of climbing across taxa and measurement of climbing performance across surfaces may illuminate new areas of interaction between morphology, performance, and fitness not discernable by quantifying clinging performance.

Future studies could also expand upon the assumptions made regarding salamander body morphology and its relationship with clinging performance. While foot shape and foot area have been the focus of research as the medium of attachment on smooth surfaces, the relationship between foot area, attachment strength, and clinging performance is multifaceted. While clinging to smooth surfaces, many salamander species use ventral portions of the trunk, tail, and lower jaw in addition to their feet to increase their total contact area with the surface (O’Donnell & Deban, 2020a). Furthermore, contact area may not be the only part of the attachment process. Salamander‐to‐substrate attachment is mediated by a layer of mucus coating the body in all salamanders. This mucus is critical in lungless plethodontid species which require a moist skin surface for the diffusion of oxygen to their tissues. Variation in the chemical makeup of salamander mucus, and its resulting material properties, might occur due to differing environmental conditions and selection for resistance to desiccation, for maximizing oxygen diffusion across the skin surface, or for predator deterrence. Determining the adhesive strength of salamander mucus and whether it varies by species, performance, or microhabitat will require additional study, but could provide further context for our results.

Overall, this study is an important example of how the ecomorphological paradigm can help explain surprising patterns or the lack thereof. The nonsignificant relationship between landmark‐based geometric morphometric quantification of foot shape and clinging performance makes plain why we do not see foot shape specialization in arboreal salamanders. That clinging performance also does not vary across microhabitat type further explains why this trait would not be under special selection based on microhabitat type. Our results reveal that, rather than foot shape, mass‐corrected foot centroid size and FCA confer variation in clinging ability and are thus more appropriate traits to expect to be under evolutionary pressures in this system. Further, the variation in performance gradients across substrate types demonstrates clearly that morphology is often under myriad selective pressures, and the ecomorphological paradigm is a valuable tool for those seeking to disentangle how complex traits relate to their ecology. Even with these exciting conclusions, many questions regarding salamander microhabitat use, clinging ability, and morphology remain unanswered: What allowed certain lineages to colonize the arboreal microhabitat? What caused many more lineages to subsequently recolonize the terrestrial microhabitat? What physiological or microscopic characteristics of salamander skin affects clinging ability? What ecological scenarios resulted in the evolution of such diverse foot shapes observed today? Future work that continues to examine how morphology, performance, and ecology relate on a macroevolutionary scale is sure to bring about answers to these and many other interesting evolutionary questions.

## CONFLICT OF INTEREST

None declared.

## AUTHOR CONTRIBUTIONS

**Erica K. Baken:** Conceptualization (lead); data curation (equal); formal analysis (lead); investigation (lead); resources (equal); writing–original draft (lead); writing–review and editing (lead). **Mary Kate O’Donnell:** Conceptualization (supporting); data curation (equal); resources (equal); writing–review and editing (supporting).

## Supporting information

Table S1

## Data Availability

All data in this manuscript are available on DRYAD (https://doi.org/10.5061/dryad.05qfttf34).
