# Supplementary material for: Clinging ability is related to particular aspects of foot morphology in salamanders
Source: Ecol Evol. 2021 Jul 17;11(16):11000–8. doi: 10.1002/ece3.7888 (PMC8366850; doi:10.1002/ece3.7888)
Supplement: Supplementary file 1 — Table S1 [file ECE3-11-11000-s001.docx]

| **Substrate** | **Term** | **DF** | **SS** | **MS** | **R^2^** | **F** | **Z** | **P Value** |
| --- | --- | --- | --- | --- | --- | --- | --- | --- |
| Smooth | **CS/Mass** | **1** | **72.464** | **72.464** | **0.431** | **8.645** | **2.051** | **0.011** |
|  | Microhabitat | 2 | 18.094 | 9.047 | 0.108 | 1.079 | 0.301 | 0.393 |
|  | (CS/Mass)*Microhabitat | 2 | 10.431 | 5.216 | 0.062 | 0.622 | -0.147 | 0.553 |
|  | Residuals | 8 | 67.061 | 8.383 | 0.399 |  |  |  |
|  | Total | 13 | 168.050 |  |  |  |  |  |
|  | **FSA/Mass** | **1** | **85.319** | **85.319** | **0.508** | **10.605** | **2.222** | **0.008** |
|  | Microhabitat | 2 | 13.145 | 6.573 | 0.078 | 0.817 | 0.040 | 0.491 |
|  | (FSA/Mass)*Microhabitat | 2 | 5.226 | 2.613 | 0.031 | 0.325 | -0.590 | 0.731 |
|  | Residuals | 8 | 64.360 | 8.045 | 0.383 |  |  |  |
|  | Total | 13 | 168.050 |  |  |  |  |  |
| Rough | **CS/Mass** | **1** | **20.408** | **20.408** | **0.285** | **9.156** | **2.023** | **0.020** |
|  | Microhabitat | 2 | 7.871 | 3.936 | 0.110 | 1.766 | 0.765 | 0.237 |
|  | **(CS/Mass)*Microhabitat** | **2** | **25.449** | **12.725** | **0.356** | **5.709** | **2.058** | **0.019** |
|  | Residuals | 8 | 17.831 | 2.229 | 0.249 |  |  |  |
|  | Total | 13 | 71.558 |  |  |  |  |  |
|  | **FSA/Mass** | **1** | **24.125** | **24.125** | **0.337** | **10.886** | **2.110** | **0.010** |
|  | Microhabitat | 2 | 6.491 | 3.245 | 0.091 | 1.465 | 0.580 | 0.265 |
|  | **(FSA/Mass)*Microhabitat** | **2** | **23.214** | **11.607** | **0.324** | **5.238** | **1.960** | **0.027** |
|  | Residuals | 8 | 17.729 | 2.216 | 0.248 |  |  |  |
|  | Total | 13 | 71.558 |  |  |  |  |  |
